# Impact of Rhabdomyolysis on Clinical Outcomes in Patients With Acute Myocardial Infarction

**DOI:** 10.7759/cureus.87142

**Published:** 2025-07-01

**Authors:** Yousef Alsmairat, Montaser Elkholy, George G Kidess, Zijin Lin, Ryan Berry, Yasemin Bahar, Alaa Diab, Timir Paul, M. Chadi Alraies

**Affiliations:** 1 Internal Medicine, Wayne State University School of Medicine, Detroit, USA; 2 Internal Medicine, Detroit Medical Center/Sinai Grace Hospital/Wayne State University, Detroit, USA; 3 Medicine, Wayne State University School of Medicine, Detroit, USA; 4 Cardiology, Michigan State University, East Lansing, USA; 5 Family Medicine, Authority Health, Detroit, USA; 6 Internal Medicine, Detroit Medical Center, Detroit, USA; 7 Section of Interventional Cardiology, University of Tennessee at Nashville/Ascension Saint Thomas Hospital, Nashville, USA; 8 Cardiology, Detroit Medical Center Heart Hospital, Detroit, USA

**Keywords:** acute kidney injury, acute myocardial infarction, in-hospital mortality, nationwide inpatient sample (nis), rhabdomyolysis

## Abstract

Background: Acute myocardial infarction (AMI) is a major cause of death and disability worldwide. The clinical outcomes of the co-occurrence of rhabdomyolysis and AMI are not very well studied. The aim of this study is to analyze the impact of rhabdomyolysis on the clinical outcomes in patients with AMI.

Methods: Between 2018 and 2021, patients diagnosed with AMI and rhabdomyolysis were identified using the National Inpatient Sample and ICD-10 codes. A multivariate regression analysis was conducted using STATA software (Stata Corp., College Station, TX).

Results: A total of 2,467,290 hospitalizations diagnosed with AMI were identified. Of those, 17,800 had a co-diagnosis of rhabdomyolysis. Compared to patients with AMI alone, patients with AMI and rhabdomyolysis had a higher in-hospital mortality rate (2,965 (16.65%) vs. 113,455 (4.63%), adjusted odds ratio (aOR) 2.38, p<0.0001), cerebrovascular accidents (CVAs) (740 (4.2%) vs. 30,740 (1.25%), aOR 2.13, p<0.0001), cardiogenic shock (3,530 (19.83%) vs. 169,490 (6.92%), aOR 2.24, p<0.0001), acute kidney injury (AKI) (10,660 (59.89%) vs. 503,815 (20.57%), aOR 5.13, p<0.0001), need for hemodialysis (1,105 (6.2%) vs. 85,160 (3.48%), aOR 1.94, p<0.0001), and prolonged length of stay (LOS) (aOR 1.98, p<0.0001). There was no statistical significance between the two groups in developing acute heart failure (4,765 (26.77%) vs. 4,899 (20.9%), aOR 0.92, p=0.112). Additionally, patients with rhabdomyolysis and AMI were less likely to undergo percutaneous coronary intervention (3,240 (18.2%) vs. 786,286 (32.1%), p<0.0001).

Conclusion: Patients who had AMI with rhabdomyolysis had substantially worse in-hospital outcomes, including higher in-hospital mortality rates, higher risks of developing CVAs, cardiogenic shock, AKI, hemodialysis, and longer LOS.

## Introduction

Rhabdomyolysis is characterized by skeletal muscle breakdown and release of intracellular contents, including myoglobin, creatinine kinase (CK), and electrolytes. Rhabdomyolysis can be a result of a traumatic injury, including crush injury or overexertion; non-traumatic etiologies, such as tissue hypoperfusion; and exposure to toxic substances [1]. Its clinical implications vary depending on the extent of the muscle injury and the organ systems affected. Cardiovascular complications can stem from electrolyte imbalances and the possible direct injury of myoglobin on blood vessels, ranging from arrhythmia to cardiac arrest [2,3].

Acute myocardial infarction (AMI) is a major cause of death and disability worldwide. It is characterized by compromised blood supply leading to myocardial cell death due to prolonged ischemia. Myocardial injury can lead to a wide range of complications affecting multiple organ systems. Cardiovascular complications can be categorized into inflammatory, embolic, and mechanical complications, including ventricular rupture, aneurysm, and cardiogenic shock [4,5].

The occurrence of AMI in the setting of rhabdomyolysis could be a result of skeletal muscle hypoperfusion and ischemia or due to mutual risk factors, such as electrolytes and metabolic disorders. This study aims to evaluate the role of rhabdomyolysis in the clinical outcomes of patients hospitalized with AMI. Previous research has extensively studied the poor clinical outcomes of rhabdomyolysis, but its effects on the clinical outcomes in patients with AMI have not been studied. To our knowledge, this is the first study that investigates the in-hospital outcomes and the interplay between rhabdomyolysis and AMI.

## Materials and methods

Study design data source

A retrospective cohort analysis was conducted using the National Inpatient Sample (NIS) from 2018 to 2021, the largest publicly available all-payer inpatient healthcare database in the United States (US). The NIS is managed by the Healthcare Cost and Utilization Project (HCUP), supported by the Agency for Healthcare Research and Quality (AHRQ). Data are stratified to identify a 20% weighted sample to represent the national sample from different hospitals and geographic locations. It includes information on patient demographics, insurance coverage, admission and discharge status, length of stay (LOS), and hospital-level information from non-federal short-term US hospitals.

Study population, study variables, and outcomes

Patients aged 18 years or older with a principal diagnosis of AMI were identified. The final analytic cohort included two groups: patients with AMI without coexisting rhabdomyolysis (Group 1) and patients with AMI and coexisting rhabdomyolysis (Group 2). These groups were compared to evaluate baseline characteristics and outcomes.

Age, sex, race, insurance type, and comorbid conditions such as chronic kidney disease (CKD), diabetes mellitus (DM), hypertension, and others were included. The history of smoking and alcohol use disorder was also assessed. The primary outcome was in-hospital mortality, whereas the secondary outcomes included acute kidney injury (AKI), acute heart failure (AHF), cardiogenic shock, LOS, and total hospitalization charges.

We identified AMI using the ICD-10 code I21 and its subcategories, which include both ST-elevation and non-ST-elevation myocardial infarctions. These codes are assigned by hospital coding professionals based on the full clinical documentation, including symptoms, ECG findings, biomarker trends, and imaging results, as applicable. This method reflects real-world administrative coding practices and is widely used in NIS.

Statistical analysis

Statistical analyses were performed using STATA version 18.5 (Stata Corp., College Station, TX). STATA facilitates analysis to produce nationally representative, unbiased results, variance estimates, and p-values. We used multivariate logistic regression analysis to adjust for confounders. The variables associated with a p-value of 0.2 on univariate logistic regression analyses were used to generate a multivariate logistic regression model. Variables found significant in the literature search were included in the final model.

## Results

The study included a total of 2,467,290 hospitalizations with a diagnosis of AMI. Of these, 17,800 (0.72%) had a co-diagnosis of rhabdomyolysis. The baseline characteristics of patients with and without rhabdomyolysis are summarized in Table 1.

**Table 1:** Baseline characteristics of AMI patients with and without coexisting rhabdomyolysis. Values are presented as numbers (percentages) unless otherwise indicated. p-values were calculated using survey-weighted chi-square tests (design-based F tests) for categorical variables and survey-weighted linear regression for continuous variables. Test statistics are reported as F-values for categorical variables and t-values for continuous variables. Those tests assess the independence of variables across the two groups. AMI, acute myocardial infarction; CKD, chronic kidney disease; GFR, glomerular filtration rate; PCI, percutaneous coronary intervention

	AMI without coexisting rhabdomyolysis (N=2,449,490) (99.27%)	AMI with coexisting rhabdomyolysis (N=17,800) (0.72%)	Test statistics	p-value
Age (mean ± SD)	49.92±27.26	68.78±15.40	t=7.82	p=0.0002
18–44 y, n (%)	128,000 (5.23%)	1,225 (6.83%)	F (2,31797) = 29.78	p<0.0001
45–64 y, n (%)	914,262 (37.38%)	5,696 (32%)	F (2,31797) = 29.78	p<0.0001
≥65 y, n (%)	1,396,228 (56.95%)	10,472 (61.12%)	F (2,31797) = 29.78	p<0.0001
Sex				
Female, n (%)	904,280 (36.93%)	6,332 (35.67%)	F (1,15902) = 2.45	p=0.1176
Male, n (%)	1,545,210 (63.07%)	11,468 (64.33%)	F (1,15902) = 2.45	p=0.1176
White	1,734,097 (70.81%)	11,588 (65.31%)	F (2.98, 47394) = 22.07	p<0.0001
African American	269,444 (11%)	2,734 (15.11%)	F (2.98, 47394) = 22.07	p<0.0001
Hispanic	216,141 (8.81%)	1,664 (9.35%)	F (2.98, 47394) = 22.07	p<0.0001
Other	229,969 (9.37%)	1,818 (10.22%)	F (2.98, 47394) = 22.07	p<0.0001
Diabetes mellitus	1,007,420 (41.19%)	5,800 (32.61%)	F (1,15902) = 107.08	p<0.0001
Hypertension	970,661 (39.57%)	4,710 (26.29%)	F (1,15902) = 244.18	p<0.0001
Smoking	1,207,620 (49.33%)	6,850 (38.48%)	F (1,15902) = 161.17	p<0.0001
Hyperlipidemia	1,574,898 (64.31%)	8,013 (46.21%)	F (1,15902) = 482.42	p<0.0001
Obesity	536,628 (21.94%)	2,835 (15.93%)	F (1,15902) = 71.82	p<0.0001
Heart failure	889,080 (36.26%)	8,073 (45.42%)	F (1,15902) = 122.35	p<0.0001
CKD (GFR<60)	366,016 (14.97%)	2,645 (14.86%)	F (1,15902) = 0.03	p=0.8591
Anemia	562,711 (22.96%)	5,998 (33.74%)	F (1,15902) = 228.38	p<0.0001
Sepsis	47,756 (1.95%)	1,745 (9.8%)	F (1,15902) = 1056.49	p<0.0001
Alcohol use	81,825 (3.34%)	1,586 (8.9%)	F (1,15902) = 328.32	p<0.0001
Cocaine use	5,147 (0.21%)	36 (0.2%)	F (1,15902) = 0.04	p=0.8397
PCI	786,286 (32.1%)	3,240 (18.2%)	F (1,15902) = 19.75	p<0.0001
Insurance status				
Medicaid, n (%)	1,423,480 (58.13%)	11,161 (62.79%)	F (3,47630) = 24.56	p<0.0001
Medicare, n (%)	248,081 (10.14%)	2,115 (11.88%)	F (3,47630) = 24.56	p<0.0001
Private, n (%)	654,367 (26.74%)	3,629 (20.41%)	F (3,47630) = 24.56	p<0.0001
Self-pay, n (%)	122,257 (4.99%)	878 (4.93%)	F (3,47630) = 24.56	p<0.0001

Demographic characteristics

There was a significant difference in the age distribution between the two groups (p<0.0001). Individuals with AMI and rhabdomyolysis were more likely to be older, with 61.12% aged 65 years or older, compared to 56.95% of patients without rhabdomyolysis. The sex distribution did not differ significantly between the two groups (p=0.1176), with similar proportions of males and females. Race showed significant differences, with a higher proportion of African American and Hispanic individuals in the AMI with rhabdomyolysis group (p<0.0001).

Comorbidities

**Figure 1:**
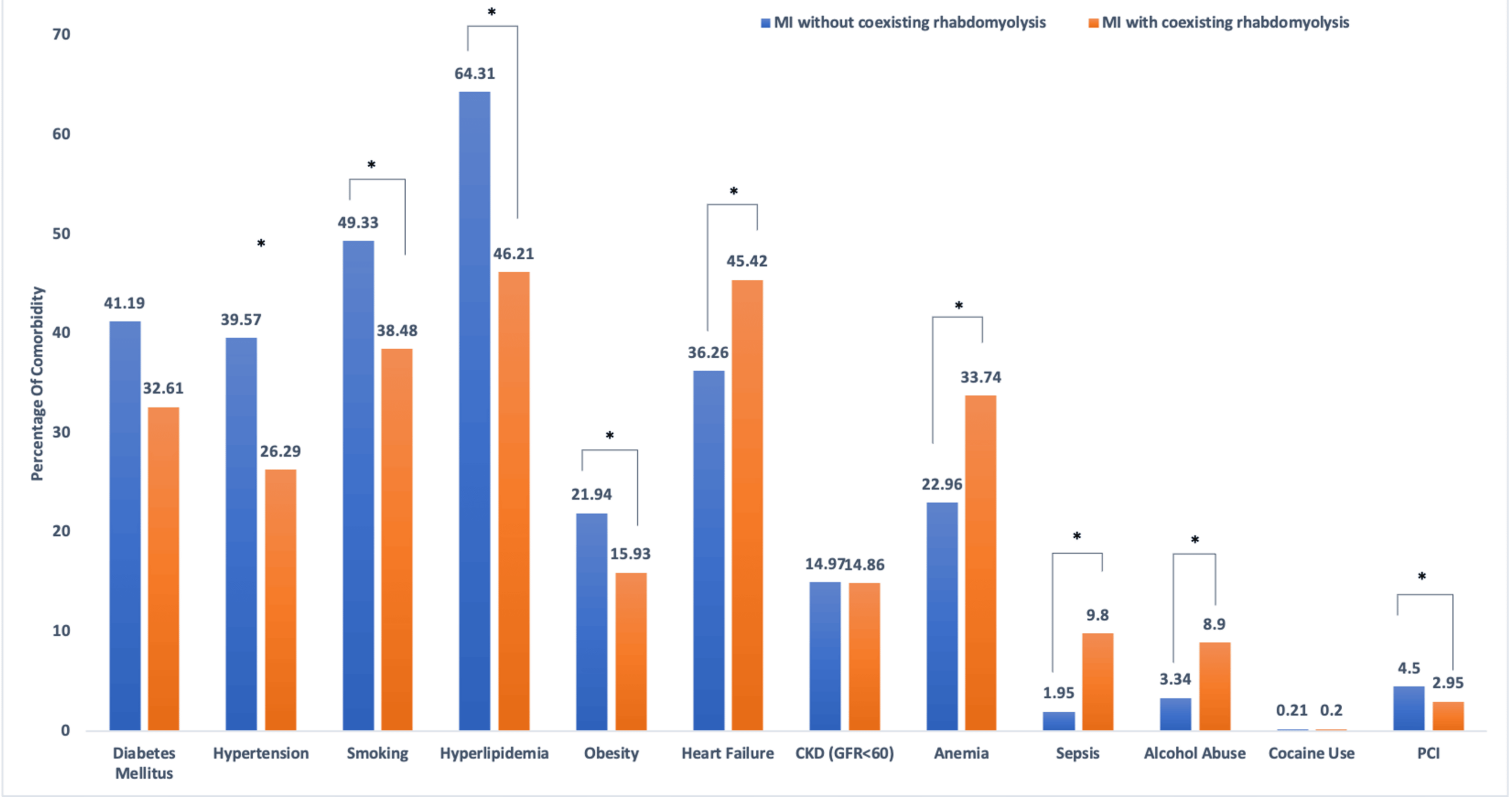
Comparison of baseline comorbidities between patients with acute MI with and without coexisting rhabdomyolysis. This bar chart illustrates the percentage of patients with selected comorbid conditions among those admitted with MI and coexisting rhabdomyolysis (orange bars, n=17,800) compared to those without rhabdomyolysis (blue bars, n=2,449,490). Comorbidities analyzed include diabetes mellitus, hypertension, smoking, hyperlipidemia, obesity, heart failure, CKD (with GFR <60 mL/min/1.73 m²), anemia, sepsis, alcohol abuse, cocaine use, and PCI. Statistically significant differences between the two groups are denoted by an asterisk (*), with MI + rhabdomyolysis patients showing higher rates of heart failure, anemia, sepsis, and substance abuse (alcohol and cocaine) but lower rates of diabetes, hypertension, smoking, and hyperlipidemia. The distribution of CKD and PCI was similar between the groups. These findings highlight distinct comorbidity profiles among MI patients with versus without concurrent rhabdomyolysis, suggesting differences in baseline health status and risk exposures. CKD, chronic kidney disease; GFR, glomerular filtration rate; MI, myocardial infarction; PCI, percutaneous coronary intervention

The prevalence of several comorbidities differed significantly between the groups. Individuals without rhabdomyolysis had higher rates of DM (1,007,420 (41.19%) vs. 5,800 (32.61%), p<0.0001), hypertension (970,661 (39.57%) vs. 4,710 (26.29%), p<0.0001), smoking (1,207,620 (49.33%) vs. 6,850 (38.48%), p<0.0001), hyperlipidemia (1,574,898 (64.31%) vs. 8,013 (46.21%), p<0.0001), and obesity (536,628 (21.94%) vs. 2,835 (15.93%), p<0.0001). In contrast, individuals with AMI and rhabdomyolysis had higher rates of heart failure (HF) (8,073 (45.42%) vs. 889,080 (36.26%), p<0.0001), anemia (5,998 (33.74%) vs. 562,711 (22.96%), p<0.0001), sepsis (1,745 (9.8%) vs. 47,756 (1.95%), p<0.0001), and alcohol use disorder (1,586 (8.9%) vs. 81,825 (3.34%), p<0.0001). There was no significant difference in the prevalence of CKD (2,645 (14.86%) vs. 366,016 (14.97%), p=0.8591) or cocaine use (36 (0.2%) vs. 5,147 (0.21%), p=0.8397) between both groups (Figure 1).

Procedural and insurance data

Individuals with AMI and rhabdomyolysis were less likely to undergo percutaneous coronary intervention (PCI) (18.2% vs. 32.1%, p<0.0001). Insurance status also differed significantly; a higher proportion of individuals with AMI and rhabdomyolysis had Medicaid coverage (11,161 (62.739%) vs. 1,423,480 (58.13%), p<0.0001), while those without rhabdomyolysis were more likely to have private insurance (654,367 (26.74%) vs. 3,629 (20.41%), p<0.0001). The proportion of individuals with self-pay status did not differ significantly between the two groups (122,257 (4.99%) vs. 878 (4.93%), p=0.8541).

In conclusion, differences between the two groups were statistically significant for age, race, comorbidities (DM, hypertension, smoking, hyperlipidemia, obesity, HF, anemia, sepsis, and alcohol abuse), PCI, and insurance status (all p<0.0001). No significant differences were found for gender, cocaine use, or CKD (p>0.05).

In-hospital outcomes

After adjusting for baseline characteristics, patients with AMI and rhabdomyolysis had higher odds of inpatient mortality, cerebrovascular accidents, cardiogenic shock, AKI, and the need for hemodialysis compared to those without rhabdomyolysis. They also experienced longer hospital stays and higher total hospital charges. There were no significant differences in the rates of AHF or PCI-related hemorrhage between the groups (Tables 2, 3 provide full statistical details).

**Table 2:** aOR and 95% CI for in-hospital outcomes in patients with AMI with and without coexisting rhabdomyolysis. This figure summarizes the results of multivariate survey-weighted logistic regression models evaluating the association between coexisting rhabdomyolysis and in-hospital outcomes among patients admitted with AMI. Models were adjusted for demographic and clinical covariates, including race, age, sex, chronic heart failure, chronic kidney disease, hyperlipidemia, obesity, coronary artery disease, smoking, hypertension, diabetes, insurance type, substance use (alcohol, cocaine), anemia, and sepsis. Test statistics are reported as design-based F statistics derived from the survey-adjusted logistic models. AKI, acute kidney injury; AMI, acute myocardial infarction; aOR, adjusted odds ratios; PCI, percutaneous coronary intervention

In-hospital outcomes	aOR	95% CI	Test statistics	p-value
Mortality	2.38	2.14–2.66	F (21,15882) = 1040.12	p<0.0001
Cerebrovascular accidents	2.13	1.78–2.56	F (1,15902) = 240.27	p<0.0001
Cardiogenic shock	2.24	2.02–2.48	F (21,15882) = 1024.79	p<0.0001
AKI	5.13	4.73–5.56	F (1,15902) = 3195.45	p<0.0001
Hemodialysis	1.94	1.57–2.40	F (1,15902) = 77.91	p<0.0001
Length of stay	1.98	1.55–2.52	F (21,15882) = 209.67	p<0.0001
Acute heart failure	0.92	0.83–1.02	F (1,15902) = 70.65	p=0.112
PCI-related hemorrhage	0.28	0.03–2.06	F (20,15883) = 14.68	p=0.214

**Table 3:** In-hospital outcomes in patients with AMI with and without coexisting rhabdomyolysis. This table summarizes the in-hospital clinical outcomes for patients admitted with AMI, stratified by the presence or absence of coexisting rhabdomyolysis. Of the total cohort (N=2,467,290), 17,800 patients (0.72%) had a concurrent diagnosis of rhabdomyolysis, while the remaining 2,449,490 patients (99.27%) had AMI without rhabdomyolysis. All statistical analyses were conducted using STATA 18.0. Categorical variables, including in-hospital outcomes, such as mortality, cerebrovascular accidents, cardiogenic shock, AKI, need for hemodialysis, acute heart failure, and PCI-related hemorrhage, were presented as counts and percentages. Differences in proportions between patients with AMI and coexisting rhabdomyolysis and those without rhabdomyolysis were assessed using the Pearson chi-square test. A two-sided p-value of <0.05 was considered statistically significant. No adjustments for multiple comparisons were applied, given the descriptive nature of this analysis. AKI, acute kidney injury; AMI, acute myocardial infarction; PCI, percutaneous coronary intervention

In-hospital outcomes	AMI without coexisting rhabdomyolysis (N=2,449,490) (99.27%)	AMI with coexisting rhabdomyolysis (N=17,800) (0.72%)
Mortality	113,455 (4.63%)	2965 (16.65%)
Cerebrovascular accidents	30,740 (1.25%)	740 (4.2%)
Cardiogenic shock	169,490 (6.92%)	3530 (19.83%)
AKI	503,815 (20.57%)	10660 (59.89%)
Hemodialysis	85,160 (3.48%)	1105 (6.2%)
Acute heart failure	512,680 (20.9%)	4765 (26.77%)
PCI-related hemorrhage	2015 (0.08%)	5 (0.03%)

**Figure 2:**
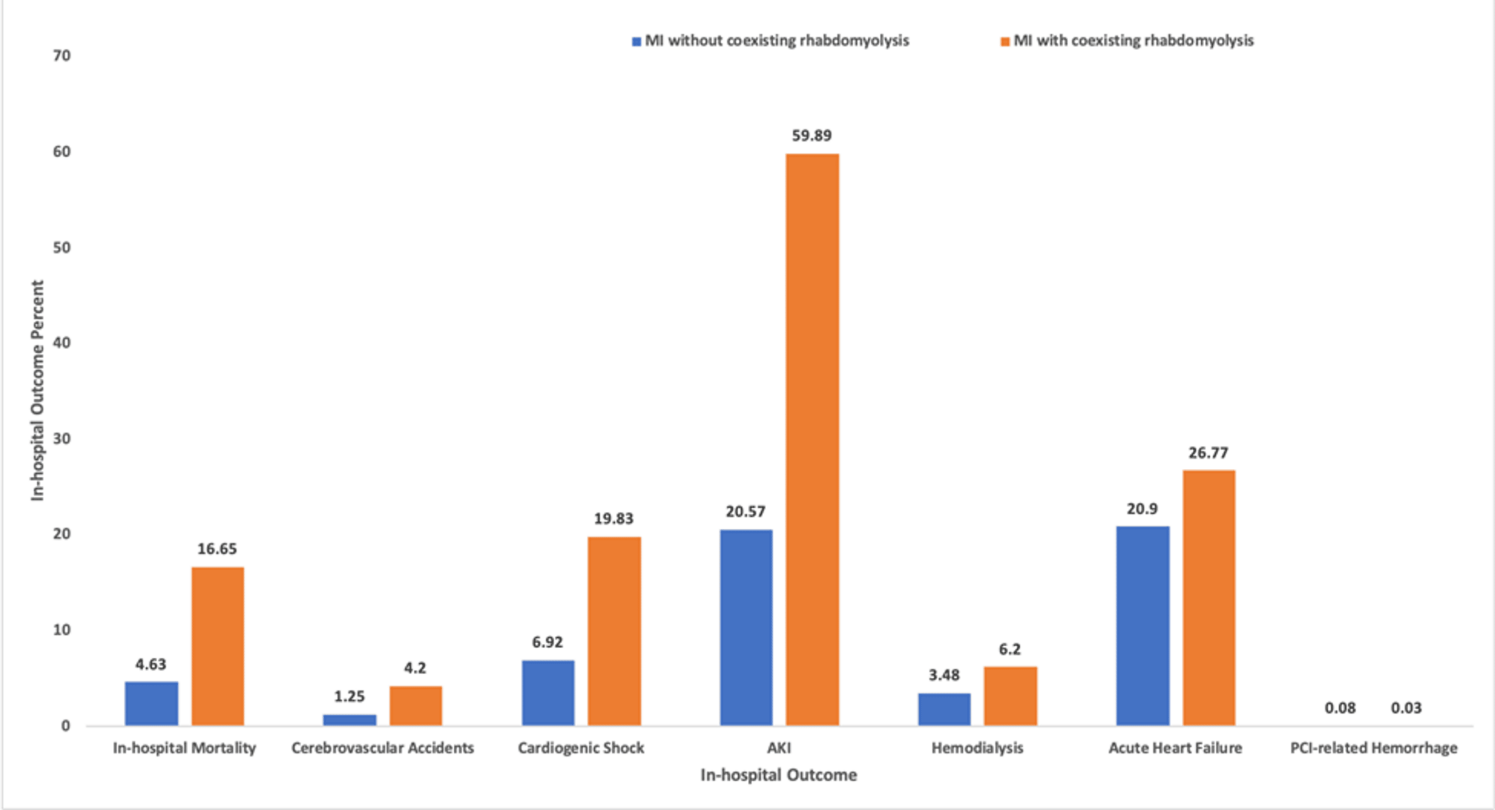
In-hospital outcomes among patients with acute MI, with and without coexisting rhabdomyolysis. This bar graph compares the percentages of major in-hospital outcomes between patients admitted with MI and coexisting rhabdomyolysis (orange bars, n=17,800) and those with MI and without rhabdomyolysis (blue bars, n=2,449,490). Outcomes assessed include in-hospital mortality, cerebrovascular accidents, cardiogenic shock, AKI, hemodialysis, acute heart failure, and PCI-related hemorrhage. Patients with MI and rhabdomyolysis experienced substantially higher rates of all adverse outcomes than those without rhabdomyolysis. Notably, mortality (16.65% vs. 4.63%), cardiogenic shock (19.83% vs. 6.92%), AKI (59.89% vs. 20.57%), and acute heart failure (26.77% vs. 0.2%) were markedly elevated in the rhabdomyolysis cohort. Rates of cerebrovascular accidents, hemodialysis, and PCI-related hemorrhage were also higher, though the latter remained low in both groups. These findings suggest that the presence of rhabdomyolysis in MI is associated with a significantly worse in-hospital clinical course. AKI, acute kidney injury; MI, myocardial infarction; PCI, percutaneous coronary intervention

Mean LOS was 1.98 times higher (95% CI: 1.55-2.52, p<0.0001) in patients with rhabdomyolysis than in those without rhabdomyolysis. AMI with coexisting rhabdomyolysis was associated with an increase in total charges of $38,992.20 on average (p<0.0001) (Table 3, Figure 2).

**Figure 3:**
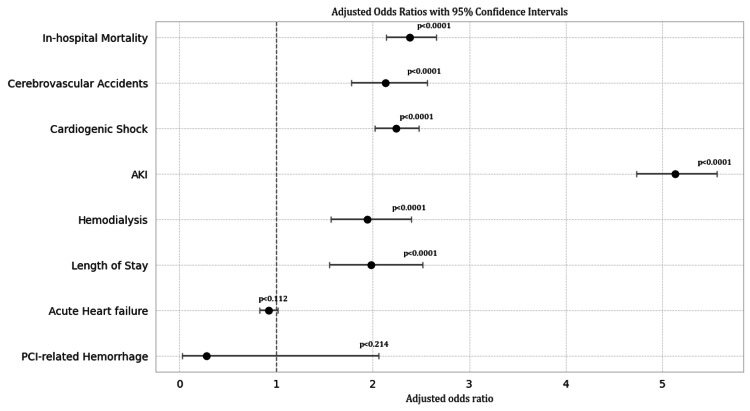
Forest plot of adjusted odds ratios with 95% confidence intervals for in-hospital outcomes in patients with MI with and without coexisting rhabdomyolysis. This forest plot displays the adjusted odds ratios and corresponding 95% confidence intervals for major in-hospital outcomes among patients admitted with MI and coexisting rhabdomyolysis compared to those without. After adjustment for demographic and clinical factors, the presence of rhabdomyolysis was associated with increased likelihood of several adverse outcomes, including death, stroke, cardiogenic shock, kidney injury, the need for dialysis, and prolonged hospital stay. No significant differences were observed in the risk of acute heart failure or bleeding related to percutaneous coronary intervention. The figure displays point estimates for each outcome along with the corresponding confidence intervals, with the vertical reference line indicating no difference between groups. MI, myocardial infarction

There was no statistically significant difference for the incidence of AHF (aOR: 0.92, 95% CI: 0.83-1.02, p=0.112) or PCI-related hemorrhage (aOR: 0.28, 95% CI: 0.03-2.06, p=0.214) between patients with and without rhabdomyolysis (Figure 3).

## Discussion

Rhabdomyolysis is a complex condition that is defined by the dissolution of damaged muscle cells, which can have a wide variety of etiologies, including ischemia, electrolyte imbalance, and medications, among others [6]. Rhabdomyolysis occurs rarely in the context of acute myocardial ischemia and has been reported in some case reports, although some uncertainty exists regarding the causal condition [7,8]. There have been some cases that report rhabdomyolysis arising after cardiopulmonary resuscitation in the context of AMI, while some experts suggest that rhabdomyolysis may also be associated with myocardial necrosis [9,10]. Given that the pathology of both diseases can be driven by tissue hypoperfusion, it is reasonable to predict that either disease can cause the other, or a separate etiology that leads to hypoperfusion can cause both diseases [6]. Evidence regarding the association of these diseases with each other and their clinical outcomes when they coexist has largely been based on case reports, and to our knowledge, no study to date has explored the clinical outcomes of patients with both diseases. Our study used the NIS to compare the in-hospital outcomes of patients admitted with both rhabdomyolysis and AMI and those admitted only with AMI.

Our study finds that patients admitted with AMI and rhabdomyolysis experience worse in-hospital outcomes with respect to several measures, including higher odds of in-hospital mortality and increased LOS. Rhabdomyolysis has been shown to increase the risk of mortality when it occurs with other diseases [11]. In patients with AMI, worse outcomes are expected, as rhabdomyolysis causes several electrolyte disturbances that can lead to multiorgan dysfunction and ultimately death [6]. One potential reason for the worse outcomes could be that patients in our study with rhabdomyolysis and AMI received PCI at significantly lower rates than those with AMI only. This can be due to the hesitancy of providers in using contrast-induced nephropathy in patients with already poor renal function secondary to rhabdomyolysis, which is associated with poorer short- and long-term outcomes [12].

This study reveals higher odds of both AKI and hemodialysis in patients with rhabdomyolysis and AMI. This is expected given that the pathophysiology of rhabdomyolysis is highly associated with renal injury, with many patients being ultimately treated with some form of renal replacement therapy [11,13]. Some studies suggest that myoglobin and hemoglobin have peroxidative effects that lead to the systemic release of vasoconstrictive agents, which have been proposed as a mechanism for AKI in this patient group [3,14]. In the AMI population, some studies showed an association between CK levels in patients experiencing ST-segment elevation myocardial infarction and AKI, although no specific mechanism was found [15]. The higher rates of kidney injury in the AMI population are significant, as it is associated with worse cardiovascular outcomes, potentially due to a higher risk of cardiac microvascular damage [16].​​ The presence of AKI is associated with widespread endothelial dysfunction, which promotes vascular instability, inflammation, and cytokine-mediated tissue injury. These proinflammatory settings may contribute to a rapid clinical deterioration, particularly in the context of acute cardiovascular events. Recent studies, including that by Prabhahar et al. [17], have emphasized the pathophysiologic role of endothelial injury in driving systemic complications in AKI, highlighting its contribution to worsened prognosis through mechanisms such as cytokine storm and impaired microvascular perfusion.

Our study also finds higher odds of CVAs associated with rhabdomyolysis and AMI. One study showed an association between CVAs and rhabdomyolysis, largely due to some patients with CVAs being immobilized, which is a risk factor for muscle compression and hypoxia, although patients who experienced both diseases had a relatively good prognosis [18]. Several proposed mechanisms exist for the association between rhabdomyolysis and CVAs. As previously mentioned, and similar to the proposed mechanism of AKI in this group, it is possible that hemoglobin and myoglobin released in rhabdomyolysis patients have peroxidative abilities, which can lead to the formation of vasoconstrictive agents linked to cerebral vasospasm [3,14]. Animal models have also found an association between systemic inflammation, neuroinflammation, and stroke, especially in the context of comorbidities that lead to a chronic inflammatory state [19]. Perhaps patients with rhabdomyolysis are at a higher risk of stroke due to both the release of vasoconstrictive agents and an increased baseline inflammatory state.

Given that vasoconstriction can be part of the pathophysiology of rhabdomyolysis and AMI, substances such as cocaine have been linked to both in few studies [20,21]; although our study did not find a significant difference in cocaine use between either group. We also find higher rates of cardiogenic shock in patients with rhabdomyolysis and AMI. Cardiogenic shock has been reported to occur in patients with rhabdomyolysis in several case reports and was suggested to carry a more morbid cardiac course, although patients generally recovered with a good prognosis [22,23]. The higher rates of cardiogenic shock in our study can also be reflective of the various other poor outcomes that were observed in patients with rhabdomyolysis and AMI. Another important finding in our study is that less than one percent of our sample had coexistent rhabdomyolysis and AMI, emphasizing how uncommon this condition can be.

Strengths

Despite the mentioned limitations, our study has several strengths. To our knowledge, this is the only study to date that explores the clinical outcomes of patients with rhabdomyolysis and AMI, showing several significant differences compared to patients admitted with lone AMI. This disparity in outcomes highlights the importance of future studies that further explore this patient population, especially in attempting to delineate temporal associations and identify specific mechanisms for the disease processes.

Limitations

Our study has several limitations that need to be acknowledged. Given that this study used data from the NIS, it is important to acknowledge the limitations inherent in using data from national databases, including the lack of a present-on-admission flag and the inability to differentiate between multiple diagnoses and multiple hospitalizations of the same diagnosis [24,25]. These limitations are especially significant in this study, as they mean that we cannot delineate temporal associations between different diagnoses, which creates difficulties in making inferences on possible causes or mechanisms of the findings of our study.

Our analysis did not include further classification of AMI subgroups, including ST-elevation and non-ST-elevation MI, which could have revealed differences in clinical outcomes and the rate of performing PCI on those patients.

 Another important limitation of our study is the absence of data on statin use and creatine kinase (CK) levels; both are clinically relevant in the context of rhabdomyolysis and AMI. Statins are a rare but known cause of rhabdomyolysis, and the inability to account for their use prevents us from assessing their potential contribution to the clinical outcomes. Similarly, CK levels are critical for confirming the diagnosis and severity of rhabdomyolysis, but they are not captured in the NIS database. We used ICD-10 codes to define rhabdomyolysis, which may lead to misclassification or underreporting bias. 

## Conclusions

Our study is the first to compare the clinical course of rhabdomyolysis and AMI patients to those with only AMI. The outcomes show that the former group has had substantially worse in-hospital outcomes, including higher in-hospital mortality rates, higher risks of developing CVAs, cardiogenic shock, AKI, hemodialysis, and longer LOS. These findings suggest that while patients with AMI and those with rhabdomyolysis have high morbidity and mortality, the combination of these conditions increases patient morbidity and mortality significantly. More studies are required to clarify the nature of these effects and to define possible approaches for enhancing the treatment of such patients. This study provides meaningful information about the characteristics and pathophysiology of the two conditions, supporting subsequent efforts to advance diagnostic accuracy, delineate risk factors, and develop effective intervention strategies in these challenging cases.

Given the significant clinical burden seen in patients with concurrent AMI and rhabdomyolysis, future research should prioritize developing clinical protocols aimed at the early detection and management of rhabdomyolysis in AMI patients. Moreover, conducting research using prospective registries with more granular data, including serial laboratory data such as CK, myoglobin levels, and renal function trends, could help establish a temporal relationship between myocardial injury and skeletal muscle breakdown. Clinical trials investigating nephroprotective strategies in this high-risk cohort may also help reduce complications associated with PCI hesitancy.

## Figures and Tables

**Figure 4:**
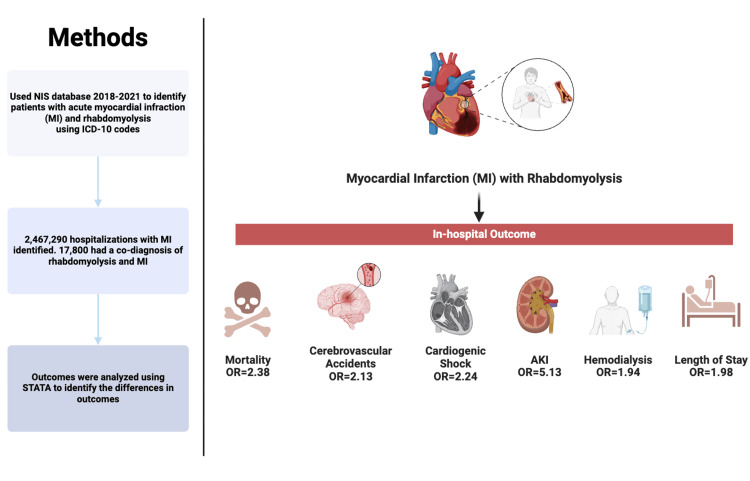
The central illustration The central illustration shows a brief description of the methods used in data extraction and analysis, including multivariate logistic regression analysis. It also shows the in-hospital outcomes with the odds ratios for patients with AMI with rhabdomyolysis.

## Appendices

**Table 4:** List of ICD-10 codes used to define diagnoses and procedures used in analysis

Diagnosis/procedure	ICD-10 code
Acute myocardial infarction	I21.0, I21.1, I21.2, I21.3, I21.4, I21.9
Rhabdomyolysis	M62.82
Chronic kidney disease	N183, N184, N185, N186
Diabetes mellitus	E10.1, E10.2, E10.3, E10.4, E10.5, E10.6, E10.8, E10.9, E11.0, E11.2, E11.3, E11.4, E11.5, E11.6, E11.8, E11.9
Cocaine use disorder	F14.90, F14.92, F14.94, F14.95, F14.98, F14.99
Hyperlipidemia	E782, E784, E785
Hypertension	I10
Tobacco use	Z72.0, Z87.891, F17.200, F17.201, F17.203, F17.210, F17.211, F17.213, F17.220, F17.221, F17.223, F17.290, F17.291, F17.293
Alcohol use disorder	F10.1, F10.2, F10.9
Heart failure	I50.1, I50.2, I50.3, I50.4, I50.9
Coronary artery disease	I25.1, I25.2, I25.3, I25.4, I25.5, I25.6, I25.7, I25.8, I25.9
Obesity	E66.01, E66.09, E66.1, E66.2, E66.8, E66.9, O99.210, O99.211, O99.212, O99.213, O99.214, O99.215, O99.280, O99.281, O99.282, O99.283, O99.284, O99.285, Z68.30, Z68.31, Z68.32, Z68.33, Z68.34, Z68.35, Z68.36, Z68.37, Z68.38, Z68.39, Z68.41, Z68.42, Z68.43, Z68.44, Z68.45, Z68.54
Anemia	D50.0, D50.1, D50.8, D50.9, D51.0, D51.1, D51.2, D51.3, D51.8, D51.9, D52.0, D52.1, D52.8, D52.9, D53.0, D53.1, D53.2, D53.8, D53.9, D55.0, D55.1, D55.2, D55.3, D55.8, D55.9, D56.0, D56.1, D56.2, D56.3, D56.4, D56.5, D56.6, D56.8, D56.9, D57.0, D57.1, D57.2, D57.3, D57.4, D57.8, D58.0, D58.1, D58.2, D58.8, D58.9, D59.0, D59.1, D59.2, D59.3, D59.4, D59.5, D59.6, D59.8, D59.9, D60.0, D60.1, D60.8, D60.9, D61.0, D61.1, D61.2, D61.3, D61.9, D62, D63.0, D63.1, D63.8, D64.0, D64.1, D64.2, D64.3, D64.4, D64.8, D64.9
Sepsis	A40.0, A40.1, A40.3, A40.8, A40.9, A41.0, A41.1, A41.2, A41.3, A41.4, A41.5, A41.8, A41.9, R65.20, R65.21, T81.12XA, T81.12XD, T81.12XS
Percutaneous coronary intervention	02700ZZ, 02703DZ, 027034Z, 027044Z, 027054Z, 02703ZZ
Acute kidney injury	N17.0, N17.1, N17.2, N17.8, N17.9
Acute heart failure	I50.21, I50.23, I50.31, I50.33, I50.41, I50.43
Hemodialysis	5A1D80Z, 5A1D90Z, 5A1D70Z
Cerebrovascular accident	I63.00, I63.01, I63.01, I63.02, I63.03, I63.09, I63.10, I63.11, I63.12, I63.13, I63.19, I63.20, I63.21, I63.22, I63.23, I63.29, I63.30, I63.31, I63.32, I63.33, I63.34, I63.39, I63.40, I63.41, I63.42, I63.43, I63.44, I63.49, I63.50, I63.51, I63.52, I63.53, I63.54, I63.59, I63.6, I63.8, I63.9
Cardiogenic shock	R57.0, T811.1XA, T811.1XD, T811.1XS
PCI-related hemorrhage	I97.610
